# Ninth International Medical Congress

**Published:** 1887-11

**Authors:** T. S. Powell


### NINTH INTERNATIONAL MEDICAL CONGRESS.

**Held in Washington, D. C., September 5, 6, 7, 8, 9 and 10, 1887.**

The session convened on Monday, September 5, at 11 o'clock, a. m., in the spacious auditorium of Albaugh's opera house. Soon as the doors were opened, the house was rapidly filled with the delegates and spectators, many ladies being among the latter.

When the curtain arose, seated upon the stage were President and Mrs. Cleveland, Secretary Bayard, Speaker Carlisle, Surgeon-General Hamilton, Dr. Garnett, members of local and other committees, and Dr. H. H. Smith, Chairman of the Executive Committee.

Dr. Smith advanced to the front of the stage, and, after calling the meeting to order, stated that in May, 1884, representative members of the profession in the United States, decided to send a fraternal greeting to the Eighth International Medical Congress, then about to assemble in the capital of Denmark and ask that the Ninth International Medical Congress meet in the City of Washington, the Capital of the United States. The invitation was accepted, an executive committee was named to make necessary arrangements, and the result of their labors was seen in that large assembly, which the register showed contained many of the most brilliant and distinguished medical minds in Europe, Asia and America.

To welcome these guests of the profession, and show his interest in a great humanitarian object, the President of the United States had consented to open the Cougress for organization, and he would have the honor of announcing the Hon. Grover Cleveland, President of the United States. When President Cleveland's name was called, the hundreds of doctors who sat so grave and dignified in their places, arose to their feet and lustily cheered, ladies waved their handkerchiefs in spontaneous enthusiasm, and the vast audience stood up and hurrahed until the building rang with prolonged cheers.

The President came forward and waited in silence, until there was a lull in the uproarious meeting, and then opened the Congress in the following brief and fitting remarks :

"I feel that the country should be congratulated to day upon the presenee at our capital of so many of our own citizens and those representing foreign countries who have distinguished themselves in the science of medicine, and are devoted to its further progress. My duty in this connection is a very pleasant and a very brief one. It is simply to declare that the Ninth International Medical Congress is now open for organization and for business." Here the President gave a stroke upon the table with a gavel, and then resumed his seat. Thus the Congress was opened.

Dr. Smith now arranged the selection of officers for the Congress by the Executive Committee.

Nathan Smith Davis, M. D., LL D., of Chicago, was unanimously chosen for the high office of President of the Congress. This grand old patriarch in medicine who has ably filled so many places of honor in the profession, is still handsomely and vigorously erect in person, and with grace and dignity took the chair, to which, at the request of Dr. Smith, he was escorted by Dr. Francisco Druante, of Rome, Italy, and Surgeon-General Marston, of England.

Dr. John B. Hamilton, of Washington, Supervising Surgeon General of the United States Marine Hospital Service, was duly elected as Secretary-General of the Congress. No better selection could have been made for the very arduous duties of the office, and which Dr. H. discharged so ably and faithfully in harmonious connection with the difficult labors performed by Dr. H. H. Smith, as Chairman of the Executive Committee.

There were about 2,500 delegates, 400 of them from foreign countries, and it is not often are assembled in association so many distinguished and fine looking men. The handsome face and genial manners of Dr. John B. Hamilton made him a pleasant and conspicuous figure throughout the convention.

The Secretary-General having announced the names of the Vice-Presidents of the Congress, the most of whom are foreign residents, the treasurer, the chairman of the Finance Committee and the associate secretaries were now nominated and elected. Then followed the nomination and election of the gentlemen who had been selected as Presidents, Vice-Presidents, and Secretaries of the several sections, after which President Davis announced the organization of the Ninth International Congress as complete, when the work of business proceeded.

Among the hundreds of professional gentlemen of the United States present, were Drs. DeLaskie Miller, of Chicago. Wm. T. Briggs, of Nashville, Dungleson, of Philadelphia, Albert L. Sitton, of United States Navy, Lewis Smith, of New York, Judson B. Andrews and Dr. W. B. Atkinson, of Philadelphia, Pennsylvania, the good old everlasting Secretary of the American Medical Association.

Also, Dr. L. Sayre, of New York; Dr. P. Sems, of Milwaukee; Dr. B. A. Watson, of Jersey City; Dr. Leartes Conner, of Detroit; Dr. Douglas Morton, of Louisville, and Dr. H. H. Mudd, of St. Louis. We note as attending from Georgia Drs. McHatton, Whitehead, Holt, Hopkins, Hobbs, Calhoun, Gaston.

The papers presented by Drs. Leon and Hobbs, with the discussion on various topics by Dr. Gaston, have received favorable notice in the published reports of the proceedings.

In alluding to these few names of those taking part in the business of different Sections, it is not intended to slight others who occupied conspicuous positions in the Congress.

In the Section of Dental and Oral Surgery many interesting papers were read, and skillful, practical illustrations made by members present; amongst those most highly complimented was Dr. Wm. Crenshaw, Professor of Operative and Clinical Dentistry in the Dental Department of the Southern Medical College, Atlanta, Ga.

Three ladies wearing the blue and silver badge of the delegates were at the opening session, and seeemed to be much interested in the proceedings.

Foreign delegates from all parts of the world represented the highest culture in medical attainments, and some of the most brilliant minds that ever illustrated the broad and noble science of the healing art.

Prominent among them were Dr. Ernest Otto, physician to King Otto, the insane young Sovereign of Bavaria, Professor Semmola, of Italy, the unequal figure of Dr. Grant, Bey of Cairo, Egypt, and a brilliant galaxy of medical lights from Great Britain, France and Germany.

Dr. Garnet announced the programme for the social entertainment of the visiting doctors and their families, the first of which was a pleasant and very largely attended *conversazione* in the magnificent hall of the United States Pension Office, on the evening of the 5th, and where the delegates had an opportunity of becoming acquainted, to give and receive fraternal greeting. The reception given the following evening by President and Mrs. Cleveland to members of the Congress and their famllies, was perhaps the most pleasing social feature of the Convention, where all were so cordially and gracefully greeted byour noble-hearted President and his lovely, charming wife. The occasion will ever remain a cherished remembrance to the visiting doctors.

There were also delightful entertainments given to the foreign delegates by citizens of Washington, a reception and grand banquet at the United States Pension Hall and excursions to Mount Vernon and Niagara Falls.

President Davis now presented to the Congress Hon. Thomas Bayard, Secretary of State, who was greeted with great applause, and in a short address of welcome in behalf of the United States, made the delegates feel at home in the beautiful Capital of the American Republic. To the Secetary's cordial and patriotic greeting, responses were given by Dr. William Henry Lloyd, of the

Royal Navy, England; Dr. Leon de Forte, of Paris, Surgeon in charge of the noted Nicker Hospital, Profesor Unna, of Germany, Professor Semmola, of Italy, and Dr. Charles Rehrer, of St. Petersburg. These were followed by the address of Dr. Davis, President of the Congress, which, as might have been expected, was eminently appropriate to the occasion, and highly suggestive of the straightforward methods and profound medical acumen of one who, during a long and honorable life, has devoted the best of himself mentally and physically to the highest interest of the profession, and its broad culture as well as its laborious work. In feeling and eloquent words, Dr. Davis first spoke of that noble and grand old man in medicine, the late Professor Austin Flint, of New York, who did more than any other to have the Ninth International Medical Congress convened in this country, but was suddenly called away from earth before he saw and enjoyed the fruition of his labors.

The Congress heard the address of the President with profound attention and absorbing interest. It was loudly applauded, and at its close, President Cleveland arose, and, approaching Dr. Davis, shook him cordially by the hand.

The Congress was divided into eighteen sections, as follows:

SECTION 1.—General Medicine. President, A. B Arnold, M. D., Baltimore, Md. Vice-Presidents, 18.

SEC. 2.—General Surgery. President, Dr. W. T. Briggs, Nashville, Tenn. Vice-Presidents, 24.

SEC. 3.—Millitary and Naval Surgery and Medicine. President, Henry Hollingsworth Smith, M. D., LL D, Philadelphia, Pa. Vice-Presidents, 32.

SEC. 4.—Obstetrics. President, Dr. DeLaskie Miller, Chicago, Ill. Vice-Presidents, 28.

SEC. 5.—Gynæcology. President, Henry O. Marcy, A. M.. M. D., LL D., Boston, Mass. Vice-Presidents, 29.

SEC. 6.—Therapeutics and Marteria Midica. President, Dr. Traill Green, Easton, Pa. Vice-Presidents, 21.

SEC. 7.—Anatomy. President, Dr. W. H. Pancoast, Philadelphia, Pa. Vice-Presidents, 98.

SEC. 8.—Physiology. President, John H. Callender, Nashville, Tenn. Vice-Presidents, 12.

SEC. 9.—Pathology. President, Professor A. B. Palmer, Ann Arbor, Mich. Vice-Presidents, 11

SEC. 10.—Diseases of Children. President, Dr. J. Lewis Smith, New York. Vice-Presidents, 26.

SEC. 11.—Ophthalmology. President, Dr. J. G. Chisholm, Baltimore, Md. Vice-Presidents, 14.

SEC. 12.—Otology. President, S. J. Jones, M. D., LL D., Chicago, Ill. Vice-Presidents, 9

SEC. 13.—Laryngology. President, W. H. Daly, M. D., Pittsburg, Pa. Vice-Presidents, 15.

SEC. 14.—Dermatology and Syphilography. President, Dr. A. R. Robinson, New York. Vice-Presidents, 16.

SEC. 15.—Public and International Hygiene. President, Dr. Joseph Jones, New Orleans. Vice-Presidents, 23.

SEC. 16.—Medical Climatology and Demography. President, Albert L. Sihon, A. M., M. D., U. S. N. Vice-Presidents, 26.

4

SEC. 17.—Psychological Medicine and Nervous Diseases. President, Judson B. Andrews, M. D., Buffalo, N. Y. Vice Presidents, 53.

SEC. 18.—Dental and Oral Surgery.—President Johathan Taft, M. D., Cincinnati, Ohio. Vice-Presidents, 12.

The general addresses by Presidents of the sections were of high character; and the various medical papers read at the daily sessions, evinced not only deep thinking and searching investigation of the subject under hand, but also gave much valuable illustration of experimental observation and practical demonstration. Our space will not permit mention of those papers in detail; but, while all were more or less valuable contributions to medical literature, some were of exceptional importance, and greatly illustrative of intelligent progress in a science of incalculable value to the human race, and of which, with all its wonderful possibilities, so little is yet positively known.

The Practice of Medince at the Present Day, was a timely and able paper by Dr. A. B. Arnold, of Baltimore. So were the papers, Typical Forms of Typhoid Fever, by Professor W. C. Dabney, of the University of Virginia.

Preventive Power of Vacination, by Dr. Joseph Korosi, of Hungary.

Conservative Obstetrics, by Professor Rodney Gleson, of Portland, Oregon.

Co'd as a Remedy in Inflammatory Affections, by Dr. Hiram, of Conshocken, Pa.; and Alcohol Organism, by Dr. Hutson Ford, of St Louis, Mo.

Some of the most valuable papers read as showing the advanceed steps in modern medical investigation of subjects of vital importance, but which, under the old *regime* of medicine, received but little or no attention, were:

The Chemical Philosophy of Medicine, by Dr. Hugh Hamilton, of Harrisburg, Pa.

The Place of Sanitary Science in Education, by Dr. A. W. Leighton, of New Haven, Conn.

Practical and Common Sense View of Public and International Hygiene, by Dr. Urial R. Milner, of New Orleans, La., and Ground Air in its Hygenic Relations, by Dr. John L. Macdonald, F. R. S. Inspector General, Royal Navy.

The Histolozy and Pathology of Reproduction, by Dr. Henry O. Marcey, of Boston, Mass., treated of a subject of the first importance.

A paper that should occupy an eminent place in the logic of medicine was read by Dr. Ouchterlong, of Louisiabia, Ky., and was entitled, The Study of the Natural History of Disease. The philosophical arguments advanced were unanswerable, and the discussions followed upon the reading of the paper were of much interest. If published in the forthcoming volumes of the Congress, we especially commend this paper to young physicians and medical students.

The general address by Dr. Marino Semmola, of Naples, Italy, on Bacteriology and its Therapeutic Relations, might well serve as a fine philosophic support to the theories advanced by Dr. Oouchterlong, and a satisfactory confirmation of the facts presented in his papeer.

All these valuable medical papers will be systematically arranged and published in several large volumes as soon as the work can be accomplished. Their contents will be of inestimable value to the profession, and no progressive, intelligent physician can afford to be withont them. The world progresses, and the physician must advance with the age in which he lives.

The great benefits to medical science and suffering humanity that will accrue from the Ninth International Medlcal Congress, lately in session at Washington, will be felt, but not known, in their full fruition by the present generation.

With but few exceptions that were finally and amicably adjusted, the harmony in the proceedings of the Congress was all that could be desired, and a feeling of general good fellowship and personal fraternity prevailed among its members.

The courtesy and hospitality of the citizens of our National Capital that was extended so cordially to the Congress, will be cherished as a most pleasant memory amid the laborious duties of the physician who finds in his work more of the prosaic than poetical aspects of life.

The Tenth International Congress will convene at Berlin in 1890, with the illustrious Veichow as President.. T. S. P.owell

---

SANDERS & SONS' EUCALYPTI EXTRACT (*Eucalyptol*).—Apply to Dr. Sander, Dillon, Iowa, for gratis supplied reports on cures effected at the clinics of the Universities of Bonn and Griefswald.

*Science* for April 29 gives its readers as a supplement two fine lithographic maps which will be of value to all who are interested in Indian history and ethnology. One of these, "The Linguistic Families of the Gulf States," defines the localities inhabited by the early Indian tribes in our Southern States. The "Town Map of the Old Creek Country" will also be of value and interest to many.

These maps are the first of a series announced some weeks ago by *Science*, and which it is proposed to issue at monthly intervals. They will be varied in character, and illustrative of geographical and political, as well as ethnological work. *Science* evidently proposes to maintain in this respect, as it has in many others, the foremost rank among American scientific journals. Published weekly, at $5 per annum, by The Science Co., 49 LaFayette Place, New York City. Sample copy free.

